# Barriers and facilitators for the implementation of a nationwide falls prevention pathway for older adults in the Netherlands

**DOI:** 10.1093/ageing/afag205

**Published:** 2026-07-13

**Authors:** Ankie de Bekker, Lidwien Lemmens, Päivi Reckman, Jolanda van der Heide, Rozan van der Veen, Judith Kuiper, Fatiha Baâdoudi

**Affiliations:** Health Services and Society, National Institute for Public Health and the Environment, Bilthoven, Netherlands; Health Services and Society, National Institute for Public Health and the Environment, Bilthoven, Netherlands; Health Services and Society, National Institute for Public Health and the Environment, Bilthoven, Netherlands; Health Services and Society, National Institute for Public Health and the Environment, Bilthoven, Netherlands; VeiligheidNL, Amsterdam, Netherlands; VeiligheidNL, Amsterdam, Netherlands; Health Services and Society, National Institute for Public Health and the Environment, Bilthoven, Netherlands

**Keywords:** falls prevention, community-dwelling, older adults, public health, cross-sectoral collaboration, healthy ageing, qualitative research

## Abstract

**Background:**

Falls contribute significantly to injury and mortality among community-dwelling older adults, with societal costs expected to rise. In the Netherlands, a nationwide falls prevention pathway, aligned with World Falls Guidelines was introduced. This includes case finding, multifactorial falls risk assessments, evidence-based interventions and sustained exercise programs. This study explores its implementation, focusing on barriers and facilitators.

**Method:**

A qualitative study using semi-structured interviews with 37 stakeholders from 11 regions in the Netherlands, including representatives from municipalities, health insurers and healthcare providers. Data were analysed using thematic analysis and guided by the Consolidated Framework for Implementation Research to explore stakeholders’ roles, intersectoral collaboration, barriers and facilitators to implementation and future perspectives.

**Results:**

Barriers include unclear role delineation, in conducting the multifactorial risk assessment, difficulties reaching high-risk older adults, and complex cross-sectoral collaboration. Limited monitoring infrastructure and inconsistent terminology hindered implementation. Facilitators included nationwide funding, regional coordination and local project leaders.

**Conclusion:**

Clearer roles, stronger healthcare-social sector partnerships and robust monitoring systems are recommended. Although substantial progress has been made, particularly in regions with pre-existing collaborative network, additional efforts are required to operationalise shared responsibility and address remaining barriers. By doing so, the pathway may enhance healthy ageing, reduce falls related injuries and alleviate the societal burden of falls.

## Key Points

- The nationwide falls prevention pathway in the Netherlands aims to reduce falls among community-dwelling older adults through case finding, multifactorial risk assessments and the delivery of evidence-based interventions.
- Effective implementation relies on cross-sectoral collaboration between healthcare and social sector, which remains challenging due to divergent priorities, professionals language and workflows.
- Strengthening partnerships, clarifying roles and responsibilities and securing sustainable funding are essential for the pathway’s long-term success.

## Introduction

Falls and their consequences significantly threaten the health and well-being of adults aged over 65 (hereafter: older adults), ranking among the leading causes of injury and mortality both in the Netherlands and globally. The World Health Organization reports that more than 172 million people suffer from short or long term injuries as a result of falls [1, 2]. In the Netherlands, falls in older adults resulted in 119,000 emergency department visits, 84,000 serious injuries (e.g. hip fracture), and 7115 deaths in 2024 [3]. These numbers are expected to rise, with the burden of disease (measured in Disability-adjusted life years (DALYs)) increasing from 127,100 DALYs in 2022 to 192,300 DALYs in 2050 [4]. Beyond immediate physical injuries, falls lead to functional, psychological and cognitive decline, reducing quality of life and often resulting in hospital admissions or long-term care placements. Falls-related medical costs in the Netherlands were 1.5 million euros in 2024 and projected to rise to 5 million by 2050 [5].

Since 2021, the Dutch government has identified falls prevention as a measure to promote healthy ageing. Healthy ageing is essential for maintaining independence and quality of life in older adults [6]. Through the national Healthy and Active Living Agreement (in Dutch: the GALA), municipalities and healthcare insurers are tasked with implementing the falls prevention pathway. GALA aims to annually identify fall risk in 14% of community-dwelling older adults and to ensure that at least 3% of this group participates in a falls prevention intervention. To support these efforts, municipalities could apply for a specific grant (SPUK funding) provided by the government. These grants are earmarked for projects and initiatives such as healthcare improvements, social projects or regional collaboration. Municipalities can allocate this funding to implement programs aimed at reducing falls among older adults and other vulnerable groups.

A national falls prevention pathway has been established. The goal is to reduce the incidence and impact of falls [5]. This pathway is in line with recommendations of the World Falls Guidelines (WFG) [7] and includes the following steps:

1) **Case finding**: identification of older adults with a fall risk based on the WFG algorithm for falls risk stratification, which provides a standardised approach to grade an individual’s risk of falling. This enables the application of proportionate, detailed assessments and interventions according to the level of risk (low, intermediate or high risk).
2) **Multifactorial falls risk assessment**: assessment for older adults identified as having a high risk of falling. This assessment identifies the individual risk factors across multiple domains. Specific tools like the Falls Analysis can be used to highlight potentially modifiable areas for intervention and facilitating a person-centred approach [8].
3) **Evidence-based falls prevention interventions**: interventions for older adults identified as having an intermediate or high risk. These are physical exercise interventions, if applicable combined with other recommended treatments or actions to reduce the risk of falls.
4) **Continuation of exercise in the community**: physical exercise activities for older adults with low risk and for individuals who have completed falls prevention interventions to ensure ongoing engagement in exercise.

**Figure 1:**
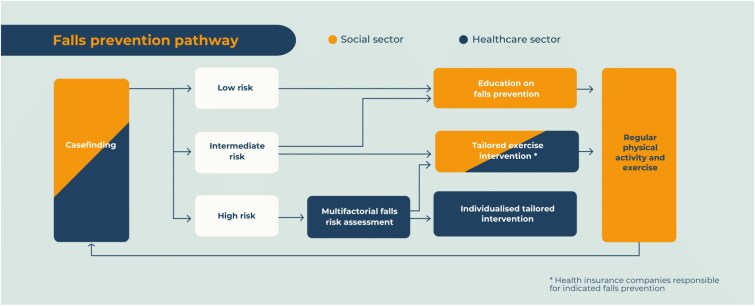
Falls prevention pathway.

Figure 1 provides an overview of the Dutch falls prevention pathway. Stakeholders operate within a decentralised healthcare system characterised by regulated competition [9]. The implementation of the falls prevention pathway is a shared responsibility of both the healthcare and social sector, necessitating cross-sectoral collaboration. This aligns with international evidence showing that successful implementation of multifactorial falls prevention interventions requires tailored strategies, stakeholder engagement and interprofessional collaboration [10]. The pathway is based on two foundations: (i) the Dutch national Health care Institute has designated the execution of falls prevention of older adults with high and intermediate risk as the responsibility of health insurance companies (healthcare sector) and (ii) the Dutch government has assigned the execution of falls prevention of older adults with low and intermediate risk as a responsibility of municipalities (social sector). For both foundations, structural financial resources have been made available. Table 1 outlines the professionals involved in each element of the falls prevention pathway.

**Table 1:** Overview of involved professionals and other stakeholders in the falls prevention programme

Element falls prevention program	Involved parties
**Case finding**	• Social workers • Domestic help • Exercise specialists • Paramedics • Healthcare professionals • Older adults themselves or their caregivers
**Multifactorial falls risk assessment**	General practitioners (GP) or medical specialists, possibly in cooperation with certified paramedics such as physiotherapists, exercise therapists or occupational therapists
**Evidence based falls prevention interventions**	Falls prevention interventions in the social sector: • Certified exercise specialists • Certified physiotherapists • Certified exercise therapists Falls prevention interventions in the healthcare sector: • Certified physiotherapists • Certified exercise therapists Other falls prevention interventions based on multifactorial falls risk assessment: • Healthcare professionals
**Continuation of regular physical activity and exercise**	Exercise specialists

Monitoring is part of the implementation of the national falls prevention pathway. Since 2023, the pathway has been monitored to provide insights into its progress, processes and outcomes. Data are collected at national level using a mixed-methods approach, incorporating both quantitative and qualitative methods. The quantitative part of the monitor includes an evaluation of the extent to which the set targets of 14% and 3% are being achieved. The qualitative part provides insight into the daily practice of implementing the pathway. The present article draws on the findings from the qualitative data collection conducted in 2024 as part of this monitoring [11, 12].

This study aims to explore the nationwide implementation of the evidence-based falls prevention pathway in the Netherlands. By analysing the barriers and facilitators experienced by key stakeholders, the study identifies factors influencing the implementation process, examines stakeholder roles and provides recommendations for improvement. This study thereby contributes to a better understanding of the practical implementation of nationwide cross-sectoral approaches in various regions, and municipalities.

## Methods

### Design

To collect information on the barriers and facilitators with the implementation of the falls prevention pathway, semi-structured interviews were conducted with stakeholders from multiple regions across the Netherlands. A predefined interview guide was used to ensure consistency across interviews. Interview topics included: participants’ roles within the falls prevention pathway, organisational agreements, experiences with (cross-sectoral) collaboration, progress of implementation process, national policy context, perceived barriers and facilitators and future plans. See Supplementary Appendix S1 in the supplementary data section for the complete interview guide. Open-ended questions were used to facilitate in-depth reflection on participants’ experiences.

### Recruitment

To ensure a comprehensive understanding of the implementation of falls prevention for community-dwelling older adults, we aimed to include a diverse sample of stakeholders in the interviews. Our goal was to capture perspectives from various parties, such as those working in the social-and healthcare sector, while also considering geographic spread, the size of the regions and the stage of implementation. Particular focus was placed on municipalities and representatives of health insurers, as they are primarily responsible for implementing the integrated approach. It should be noted, however, that fewer individual interviews were held with health insurers in absolute numbers. This is because representatives of health insurers often oversee multiple municipalities, allowing for broader insights to be gained from fewer interviews. Other stakeholders were included to ensure the incorporation of diverse perspectives. Using purposive sampling, interviewees were approached via email to participate in an interview. Also, the snow-ball technique was used to recruit interviewees. The national association for municipalities also sent a message among its members asking for potential participants. The first interviewees from a region put the researchers in touch with other relevant people in their surroundings. Inclusion criteria were stakeholders involved in the implementation of falls prevention in their region. Effort was made to ensure diversity among participants and their roles based on the aforementioned considerations. Prior to the interview, participants were contacted by one of the researchers to inform them about the purpose, procedure and confidentiality of the study. When a participant agreed on participating, an interview was arranged. Participants gave written or oral informed consent prior to the interview, including consent on the collection of their experiences, audio recording of the interviews, and the use of anonymised data for research purposes. Recruitment was considered complete when data saturation was reached.

### Data collection

Interviews were held online, by telephone or face-to-face. The duration of the interviews varied between 45 and 90 min. Interviews were audio recorded. In total, five researchers conducted the interviews. The team of researchers has experience in qualitative research (MSc or PhD) and had backgrounds in global health, public health, nursing science and health science. None of the researchers had a previous working relationship or prior research collaborations with any of the study participants. The interviews were conducted over a period of 5 months (February—June 2024).

### Analysis

**Figure 2:**
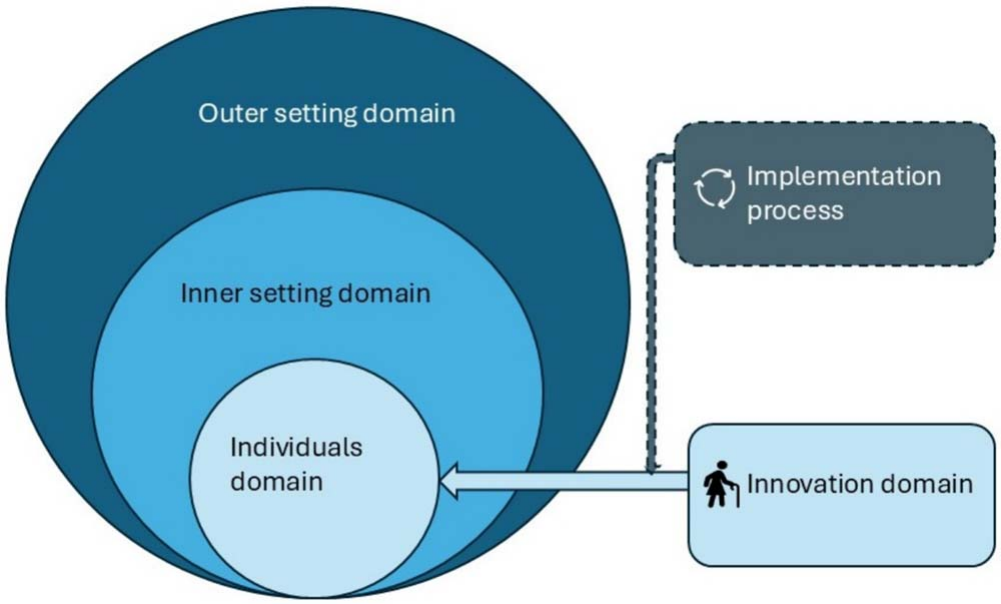
Overview of the CFIR framework, showing the interaction between individuals, organisations (inner setting), policies (outer setting), and processes.

Interviews were transcribed verbatim. The research team thematically analysed the interviews [13]. A code tree was drawn up based on the interview guide. Deriving from what the researchers found in the transcripts, the code tree was supplemented with new codes. Thematic analysis enabled the researchers to find themes and patterns within and across the data set of interviews and was mainly inductive. Transcripts from every interview were coded by two researchers using MAXQDA (version 22). Any disagreements between the two researchers were discussed to reach consensus. Participant quotes were selected during the thematic analysis process. Quotes were selected when they illustrated the perspectives of different stakeholders on key themes and provided context for the findings. The Consolidated Framework for Implementation Research (CFIR) was used to organise and interpret the results [14]. Figure 2 provides a schematic overview of how the CFIR framework works. The use of the CFIR framework provides a structured approach to identifying factors influencing implementation. These domains include: ‘the innovation’ (characteristics of the intervention), ‘outer setting’ (external factors such as policies and collaborations), ‘inner setting’ (organisational context like culture and resources), ‘characteristics of individuals’ (attitudes and skills of those involved), and ‘implementation process’ (steps and strategies for implementation). This approach helps to systematically interpret the findings.

### Ethical approval

This study was assessed by the Centre for Clinical Expertise at the RIVM (study number VPZ-609). The study did not meet the specific conditions stated in the Law for Medical Research involving Human subjects (in Dutch: Wet Medisch-Wetenschappelijk Onderzoek, WMO). Therefore, a non-WMO declaration was provided, and further ethical approval by the ethical research committee was not required.

## Results

A total of 28 interviews (individual and duo) were conducted with 37 participants across 11 municipalities. Duo interviews were conducted when participants worked closely together or shared responsibilities related to the falls prevention pathway. Interviewees included project leaders, policy advisors and professionals from municipalities, health insurers and care organisations. Table 2 provides an overview of the interviewees and their organisational or professional background. Data saturation occurred after 20 interviews, meaning no new themes emerged beyond this point. Additional interviews ensured regional representation and perspective variation.

**Table 2:** Overview of interviewees and their professional or organisational backgrounds

Affiliation or profession	*N*
Representative of municipality	13
Representative of Municipal health services (GGD)	6
Physiotherapist	6
Representative of health insurers General practitioner/Physician assistant	5 2
Exercise specialist	2
Older people association	1
Researcher	1
Welfare organisation	1
Total	37

Analysis identified 16 barriers and 13 facilitators, mapped to the CFIR framework domains. These barriers and facilitators were observed across all components of the falls prevention pathway. Sixteen constructs were identified within five broad CFIR domains. These constructs reflect the factors affecting implementation of the falls prevention pathway. Each barrier and facilitator were reviewed and grouped based on shared themes or underlying factors that influenced implementation. For example, issues related to unclear roles and cross-sectoral collaboration were grouped under the construct ‘complexity’ within the innovation domain. Results are structured by CFIR domains (Table 3).

**Table 3:** Identified barriers and facilitators for implementing falls prevention pathway, structured according to CFIR domains

CFIR domains	Constructs	Related barriers (B)/facilitators (F)
Innovation domain	Adaptability	**F**: The successful use of diverse case-finding methods supports the identification of individuals at risk.
	Complexibility	**B**: Establishing cross-sectoral collaboration is needed but also challenging and can hinder effective implementation.
	Cost	**B**: Concerns about co-payment and dissatisfaction with intervention fees impede participation.
Outer setting domain	Financing	**F**: SPUK funding enables municipalities to implement falls prevention programs
	Performance measurements	**F**: Clear goals and objectives are in the GALA agreement, provide direction for stakeholders.
	Policies and laws	**B:** A clear framework with consistent terminology is lacking, leading to confusion. **B**: Lack of clear agreements on who can execute the multifactorial falls risk assessment delays implementation.
	Financing	**B:** limited flexibility for municipalities in the allocating funding constrains local implementation efforts.
Inner setting domain	Work infrastructure	**F**: Regional collaboration supports the establishment of regional agreements and alignment of efforts.
	Physical infrastructure	**F**: The presence of sports and exercise facilities and trained intervention providers facilitates program delivery. **F**: Well-organised connections between healthcare and social sector promote coordinated implementation.
	Relational connections	**B**: Shortage of training positions for instructors limits capacity for delivering interventions.
Individuals domain	Implementation	**F**: Project leaders effectively bring organisations together and progress towards locally adapted falls prevention pathways. **F**: Awareness of the need for falls prevention is gradually increasing, supporting broader engagement.
	Recipients	**B**:: Reaching the entire target population proves difficult, reducing overall program reach.
	Capability	**B**: Professionals lack sufficient information of appropriate sports and exercise facilities for referrals.
Implementation process domain	Teaming	**F**: Stakeholders actively build relationships and engage multiple parties, strengthening collaboration. **F**: Municipalities and health insurers taking an active role in implementation. **F**: Enthusiasm and goodwill among involved parties.
	Implementation	**B**: Monitoring is limited, and the absence of adequate registration systems in the social sector hampers data-driven improvement
	Teaming	**B**: Time is needed for professionals to get to know and understand each other’s roles, delaying effective collaboration.
	Recipients	**B**: Unclear guidance and policy decisions on responsibilities of the pathway create barriers to accessing appropriate falls prevention interventions.

### Innovation domain: the innovation (Falls prevention pathway) being implemented including facilitators and barriers regarding the complex nature of the programme

#### Addressing the complexity of cross-sector collaboration

Falls prevention requires cross-sectoral collaboration, yet respondents noted that the different sectors are not communicating effectively with one another despite some coordination. For this pathway to succeed, it requires a genuinely collaborative effort and unified strategy where all sectors communicate effectively and align together.

#### Adapting methods for case finding

By employing multiple methods for case finding, interviewees aimed to reach a larger number of community-dwelling older adults, thereby increasing the likelihood of successfully identifying those at risk of falling. Case finding methods include community-based vitality events using tools like the Smart Floor^1^ and the Fall Risk Test^2^, or individual home visits by municipal or welfare staff. At the time of the interviews, it remained unclear to what extent the target group was being reached and whether the case-finding methods used were effective. However, interviewees expected that, at this stage, the majority of those identified consisted mainly of the ‘low-hanging fruit’, older adults who were already relatively easy to engage with or identify.

#### Managing costs and concerns about co-payment

Municipalities fund falls prevention in various ways, offering free, partially subsidised or refundable interventions. Many municipalities remain uncertain about the best approach, particularly regarding whether to charge participants a co-payment and, if so, how much. While co-payment could promote commitment, there are concerns they may discourage participation among older adults.

‘It should not be the case that based on income, you have more or less right to follow such a programme’ (Representative of municipality)

Municipalities also face challenges in negotiating intervention fees with physiotherapists, who are often not organised locally or regionally, requiring individual agreements. Efforts to standardise fees regionally are ongoing but time-consuming. Additionally, it remains unclear whether fees in the social sector should match those in the healthcare sector. Physiotherapists reported that healthcare sector reimbursement are below cost price, also municipalities reported lacking information about health insurers’ fees.

### Outer setting domain: the setting in which the inner setting exists, including conditions external to the implementing organisations

#### Implementing falls prevention through national funding and performance targets

The GALA agreement outlined specific goals and objectives for falls prevention, providing stakeholders with clear targets. The targets were considered as beneficial because they offered a shared objective and concrete focus for collective efforts. However, there were concerns among some interviewees as they questioned whether it would be possible to achieve the targets. Due to the allocation of funding and the formulation of responsibilities and targets, many regions initiated the implementation of the falls prevention pathway.

#### Establishing a clear framework and consistent terminology

Implementation of falls prevention was delayed by unclear processes and a lack of national frameworks. Across the country, the implementation had already started while national frameworks were still lacking. According to the interviewees, information was shared too late and changed regularly in 2023 and early 2024, making it difficult to draw up local policy. Interviewees expressed concerns about the impact this has on the implementation speed. For example, professionals had to be trained to provide falls prevention interventions, however it was not yet clear which interventions would be approved. In addition, it is considered inconvenient and confusing that different terms are used for the same elements in the falls prevention pathway. For the multifactorial falls risk assessment different terms were used such as screening, falls risk assessment and falls analysis.

#### Clarifying agreements for the multifactorial falls risk assessment

Within the falls prevention pathway, the multifactorial falls risk assessment appeared to be a major implementation bottleneck. During the interview period, there was confusion regarding the role of GPs. Stakeholders disagreed on who should perform the multifactorial falls risk assessment, with municipalities advocating flexibility and health insurers imposing stricter rules. According to interviewees, parties at the national level failed to reach clear agreements. Municipalities and health insurers often could not agree on how the multifactorial falls risk assessment should be organised. Meanwhile, physiotherapists, who expressed willingness to take on this role, lack the authority to do so under current guidelines. This limitation was seen as a missed opportunity by several stakeholders, as physiotherapists are often well-positioned to conduct such assessments due to their expertise and availability. Health insurers, on the other hand, were concerned that older people would be incorrectly indicated as having a high risk of falling, creating additional pressure and costs for the healthcare sector. This led to frustration among various parties involved, with no clear resolution in sight. As a result, some municipalities delayed implementing the multifactorial falls risk assessment.

‘And you also notice with GP practices, some are open to it, but you just notice, those GPs are completely overloaded. That remains really tricky’ (physiotherapist)

#### Increasing flexibility in the timing of spending the allocated funds

A barrier to using the funding was the restriction on transferring a limited budget from the first year 2023 to 2024. Municipalities faced time pressure to spend allocated funds. Consequently, the funds could not be utilised within the stipulated timeframe and expired, causing frustration as they could have been effectively used in the second year.

‘Every municipality was really searching in the beginning. So, we kind of raced in the last six months. We all have quite a lot of money left from falls prevention. That's really super, super unfortunate’ (representative municipality)

### Inner setting domain: the setting in which the innovation is implemented including conditions internal to the organisations that are commissioned to organise the pathway locally

#### Work infrastructure: establishing roles and responsibilities and promoting learning through regional collaboration

In addition to local collaboration, regional collaboration among municipal health services (GGD), municipalities, health insurer, healthcare providers, GPs and healthcare groups supported pathway implementation. For example, regional collaboration, that is coordinated by the GGD, helps establish agreements on organising the multifactorial risk assessment. In some areas, regional working groups have been organised where municipalities share their experiences and learn from each other. Also, these groups can facilitate discussions on which agreements should be made at the regional level and which at the local level, thereby defining corresponding roles and responsibilities. The value of such working groups varies across municipalities, with those in the early stages of implementation benefiting the most.

‘At the same time, it is challenging, because municipalities that are further along in implementing the falls prevention pathway sometimes feel like: well, why am I here?’ (representative of municipal health services)

#### Physical infrastructure: accelerating implementation by training more professionals

The interventions offered, differed per municipality. Considerations in choosing an intervention are often practical in nature, such as local expertise and availability of courses for trainers. According to the interviewees, it is beneficial having already trained exercise providers before the start of the implementation, as they can be deployed quickly. Although the interviewees reported to be pleased with the increase of training places for trainers for the falls prevention interventions and multifactorial falls risk assessment, there still appears to be a shortage of training places. This shortage hinders the implementation process and demotivates professionals.

#### Relational connections: building well-organised connections between healthcare and social sector

According to those interviewed, municipalities already offer a wide range of activities that can be integrated into the falls prevention pathway. However, the most critical need is a good connection between exercise providers in the healthcare and social sector. Most municipalities have yet to establish concrete agreements for referring older adults to sport and exercise programs or facilities after the falls prevention intervention.

### Individuals domain: the roles and characteristics of the individuals involved in the commissioning and organisation of falls prevention that influence adoption and spread of the programme

#### Advancing implementation through project leaders

Municipalities used the obtained funding to appoint a project leader. Having a local or regional project leader is seen as an important element in moving forward with implementation. The project leader is responsible for bringing relevant organisations together and progressing to a locally adapted falls prevention pathway.

#### Overcoming challenges in reaching the (whole) target group

The interviews revealed challenges in reaching the whole target group with the current case finding methods. For example, relatively vital older adults attend community vitality gatherings and older people with fragile health are not reached. Also, the 65- to 75-year-old group is often missed since home visits focus on those over 75. Furthermore, interviewees indicated that they currently do not specifically target frail older people such as those with dementia or those with low socio-economic status. At the same time, interviewees noted that awareness of fall prevention is gradually increasing under older adults.

‘I notice that it is becoming much better known. That people hear it from their neighbour or friend or sister, that they participated in falls prevention. That does work’ (Community worker, welfare organisation)

#### Capability: addressing gaps in knowledge about sport and exercise facilities

Important facilitators for implementation of the falls prevention pathway include the presence of sports and exercise facilities and awareness of these facilities. Interviewees emphasised the necessity of having sufficient sports and exercise facilities nearby, enabling community-dwelling older adults to continue exercising after the intervention. The exercise specialist is expected to be able to play an important role in helping older adults to stay active. Interviews revealed that citizens, healthcare professionals, municipalities and welfare staff often lack comprehensive knowledge of the available sport and exercise programs and facilities in their region. This hinders professionals from referring older adults to appropriate sports or exercise programs or facilities nearby.

‘Exercise specialists can have a very big, important role, but that is often not yet known to physiotherapists, for instance’ (municipal health services)

‘I had contact with the exercise specialist last week. I agreed with her that I’m going to get her involved. She is going to look with those people: what is fun for them to do in the neighbourhood? Where can they go?’ (Physiotherapist)

### Implementation process: the activities and strategies used to implement the innovation, including the challenges faced by stakeholders implementing falls prevention

#### Building relationships through collaboration and teamwork

It takes time for different organisations and professionals to get to know each other and understand each other’s terminology. In areas where cross-sectoral collaboration was already established, the falls prevention pathway could be implemented and refined more rapidly. An important promoting element was that municipalities and health insurers took an active role and responsibility to implement the falls prevention pathway. Interviewees indicated the importance of involving various parties such as municipalities, physiotherapists, occupational therapists, exercise specialists and health and welfare organisations at an early stage and meet regularly. Enthusiasm and goodwill within the various parties help the engagement in falls prevention. However, a balanced number of involved parties is warranted. The interviewees mentioned that too large a group can hinder progress.

‘I think falls prevention is a very nice vehicle because health and welfare professionals are finally really getting to know each other’ (representative of municipality)

#### Finding suitable falls prevention exercise interventions for older adults

According to some interviewees, older adults who are at high risk of falling but do not have other health issues or significant frailty may struggle to find suitable exercise interventions. Because these people do not have health problems or significant frailty, falls prevention is not covered by the health insurance. At the same time these older adults might not be suitable for a group intervention in de the social sector. Furthermore, the coordination and referral between the healthcare and social sectors are not yet fully operational. As a result, older adults are often left to navigate the system on their own, which can be challenging for them. Unclear guidance and the way the funding system is organised create barriers to accessing appropriate falls prevention interventions.

#### Addressing the limited monitoring of falls prevention

The interviews indicated that the implementation process is often not yet advanced enough to allow for reflection and evaluation of the falls prevention pathway. The primary focus has been on setting up interventions and identifying community-dwelling older adults at risk of falling. However, there are plans to initiate monitoring of the pathway in the near future. A significant barrier to monitoring is the lack of registration systems for municipalities to track falls prevention efforts.

## Discussion

This study identified 16 barriers and 13 facilitators in implementing the Dutch falls prevention pathway, offering insight into nationwide challenges and opportunities. Although 80% of municipalities having initiated implementation [15], achieving the goals of annually reaching 14% of community-dwelling older adults and 3% participation in falls prevention intervention remains challenging. Numerous obstacles persist in translating the falls prevention pathway into routine practice, thus hindering both the reach of the target population and establishment of sustainable cross-sectoral collaboration. Many of these challenges align with international findings.

### Reaching the entire target population

Reaching underserved groups, such as frail older adults and those with lower socioeconomic status, remains challenging. Similar barriers have been observed internationally, where tailored strategies, such as culturally sensitive communication and patient empowerment, have been found to improve participation [10]. The pathway may need to be adapted to better address the needs of underserved groups.

### Cross-sectoral collaboration

Cross-sectoral collaboration is essential, and existing collaborations show the value of long-term partnerships and trust [16–20]. However, collaboration can be hindered by differing methods, terminologies, priorities and fragmented communication. This is consistent with factors mentioned in previous studies [10, 21, 22]. Stakeholders emphasise the need for better communication, shared goals and streamlined processes. Local coordinators and multidisciplinary work groups enhance collaboration but are not widely adopted. Similar experiences in public health indicate that collaboration can be strengthened but requires time, stable leadership and iterative learning cycles [23].

### Multifactorial risk assessment

Unclear agreements regarding the conduct of the multifactorial risk assessment have caused delays and frustration. According to a statement from the Dutch general practitioners association (LHV), GPs lack time and capacity to perform the multifactorial falls risk assessment, despite being designated by the Dutch health care institute as a key stakeholder in this task. While national policy allows physiotherapists or occupational therapists to play a role in these assessments under GP supervision, the administrative burden of routing declarations through GPs leads to resistance. This highlights a gap between policies and practice. Lack of time to execute certain parts of the falls prevention pathway is also reported in other studies [24–26].

### Sustainability of funding

SPUK funds (specific grant for municipalities) were crucial for implementation, aligning with studies on the importance of financial initiatives [16, 27]. However, this is short term funding. This can cause uncertainty about future funding, which interferes with planning and sustainability [2].

### The Dutch falls prevention pathway in the context of the World Falls Guidelines

The World Falls Guidelines (WFG) 2022 provide global recommendations for fall prevention, including risk stratification, assessment, management and intervention strategies for older adults [7]. The Dutch falls prevention pathway aligns with WFG recommendations on structured risk assessment and evidence-based interventions. However, implementing the multifactorial fall risk assessment remains challenging in the Dutch pathway. Other countries face similar issues [28]. In the UK and Ireland, WFG adoption is limited, especially in hospitals, due to limited resources and challenges in integrating care. In the USA, WFG guidelines are seen as overly complex [29] New Zealand’s comparable falls prevention programme highlights the importance of primary care engagement, standardised protocols and public awareness campaigns, all of which have contributed to increased participation. [30]

The Dutch falls prevention pathway provides several lessons that could be valuable for other countries, particularly in its integration of national coordination with local implementation and its emphasis on cross-sectoral collaboration between healthcare and social sectors. The availability of targeted funding also provides a financial model that could inspire similar initiatives elsewhere. However, challenges, such as fragmented collaboration and difficulties in reaching underserved populations, highlight the need for continuous refinement.

### Limitations of the study

This study offers insights into the Dutch falls prevention pathway, but has methodological limitations. The study primarily focused on a limited number of regions, which may not fully capture national variability. Furthermore, although cross-sectoral collaboration was explored, the perspectives of professionals in the medical sector were somewhat underrepresented. Additionally, the perspectives of the older adults themselves were not directly included. Future research should include these perspectives to provide a more comprehensive understanding of the pathway’s impact. This could be achieved by collecting their perspectives through interviews, focus groups or surveys and using these in the further development and refinement of falls prevention pathways.

## Conclusion

The implementation of the falls prevention pathway has made considerable progress, with most municipalities having taken initial steps towards its implementation. However, key challenges persist, including fragmented cross-sectoral collaboration, inconsistent execution of the multifactorial falls risk assessment and difficulties in reaching the entire target population, especially the high-risk and vulnerable older adults. To enhance the pathway’s sustainability and effectiveness, it is essential to strengthen collaboration between the healthcare and social sector, secure structural funding, and establish robust monitoring systems. Addressing these challenges will promote healthy ageing and help to reduce the societal burden of falls.

## Supplementary Material

Supplementary_materials_afag205

## Footnotes

1 The Smart Floor is a mat equipped with sensors that measures different gate parameters. Using these parameters, an algorithm predicts the probability of falling.

2 Fall Risk Test is a decision tree enabling quick falls risk stratification, based on the algorithm of the World Guidelines for falls prevention.
