# Supplementary material for: Barriers and facilitators for the implementation of a nationwide falls prevention pathway for older adults in the Netherlands
Source: Age Ageing. 2026 Jul 13;55(7):afag205. doi: 10.1093/ageing/afag205 (PMC13362963; doi:10.1093/ageing/afag205)
Supplement: Supplementary_materials_afag205 [file supplementary_materials_afag205.docx]

**Barriers and facilitators for the implementation of a nationwide Falls Prevention Pathway for older adults in the Netherlands.**

**Appendix 1. Interview guide**

**Introduction**

- Introduction and consent.

**Opening question**

1. Can you briefly introduce yourself, your role, and how you are involved in fall prevention?

*Components of the falls prevention pathway: 1.) Identifying fall risks, 2.) Screening, 3.) Fall prevention exercise programs, 4.) Referring to regular physical activity and exercise.*

1. Which components of falls prevention pathway are part of your program in you municipality/region?

- What is the role of [your organization] in each component?
- What has been set up for this?
- Which falls prevention exercise interventions are offered in your municipality?
  1. Are these evidence-based interventions (e.g., *In Balance* (also part of TOM, *Thuis Onbezorgd Mobiel*), *Otago*, and *Vallen Verleden Tijd*)?
  2. If not, why not? Which interventions are offered instead, and why?

**Reflection on 2023**

1. Have agreements been made regarding all components (see question 1) of the falls prevention pathway?

- How were (local/regional) agreements established over the past year?
- What agreements have been made?

1. Which parties are involved, from which sector, and for which components of the falls prevention pathway?

- What are the experiences with the cross-domain collaborations?
  1. Are there integrated networks involved?
  2. Are there agreements with health insurers/physiotherapists?
- What went well, and what went less well in terms of collaboration or establishing collaboration?
  1. Within the municipality/social domain and/or healthcare domain?

1. To what extent have these agreements already been implemented, and how is that progressing?

- Which components have been outsourced, to whom, and why?
- Which components have not yet started, and why not?

1. Has a lead or coordinator been appointed for the falls prevention pathway?

- Who is the lead within the municipality/social domain, and who within the healthcare domain?
- Are these leads also involved in one or more of the other chain approaches?

1. What agreements have been made with the health insurer about guiding older adults toward fall prevention exercise interventions in the social and healthcare domains?

- How is this being implemented in practice?

1. Have specific agreements or plans been made to reach vulnerable groups of older adults who are typically harder to engage in fall prevention programs?

- What are these agreements?
- How are these agreements working in practice?

1. Were there any prior agreements, plans, or activities related to falls prevention?

- What has changed since the introduction of the falls prevention pathway and GALA/SPUK?

1. Is the falls prevention pathway connected to other chain approaches or activities/interventions in the field of preventive elder care (under the Public Health Act (WPG) or Social Support Act (WMO)) or themes like active aging (GALA/SPUK), or activities in the context of WOZO or IZA?

- If so, which activities or interventions?

1. What lessons can be learned based on the experiences with the falls prevention pathway?

- What facilitated implementation? What hindered it?
- What else is needed to initiate the intended shift toward more integrated policy and cross-domain collaboration?

**Looking ahead to 2024**
12. What are the agreements and plans for the short term (2024) regarding the implementation of the falls prevention pathway?

- Have agreements been made about monitoring? If so, what are they?

1. What are the agreements and plans for the longer term?
2. How and within what timeframe will plans to reach vulnerable groups of older adults be executed?
3. How will the falls prevention pathway be secured and sustained within the municipality/region?
4. Are there other stakeholders we should speak with regarding the chain approach in your municipality? For example, a neighbourhood sports coach or physiotherapist.

- If so, who? Could you share their name and email address with us?

**Thank you!**
